# Efficacy of colistin-based combinations against pandrug-resistant whole-genome-sequenced *Klebsiella pneumoniae* isolated from hospitalized patients in Egypt: an in vitro/vivo comparative study

**DOI:** 10.1186/s13099-024-00667-z

**Published:** 2024-12-03

**Authors:** Eriny T. Attalla, Amal M. Khalil, Azza S. Zakaria, Rhiannon Evans, Nesrin S. Tolba, Nelly M. Mohamed

**Affiliations:** 1 Microbiology and Immunology Department, Faculty of Pharmacy, Alexandria University, Alexandria, Egypt; 2 Quadram Institute Bioscience, Norwich, UK; 3 Pathology Department, Medical Research Institute, Alexandria University, Alexandria, Egypt

**Keywords:** *Klebsiella pneumoniae*, Pandrug-resistant, Intensive care units, Egypt, Colistin resistance, Whole-genome sequencing, Combination therapy, Doxycycline, Levofloxacin, In vivo murine model

## Abstract

**Background:**

Colistin resistance significantly constrains available treatment options and results in the emergence of pandrug-resistant (PDR) strains. Treating PDR infections is a major public health issue. A promising solution lies in using colistin-based combinations. Despite the availability of in vitro data evaluating these combinations, the in vivo studies remain limited.

**Results:**

Thirty colistin-resistant *Klebsiella pneumoniae* (ColRKp) isolates were collected from hospitalized patients. Colistin resistance was detected using broth microdilution, and antimicrobial susceptibility was tested using the Kirby-Bauer method against 18 antibiotics. Extremely high resistance levels were detected, with 17% of the isolates being PDR. Virulence profiling, assessed using Anthony capsule staining, the string test, and the crystal violet assay, indicated the predominance of non-biofilm formers and non-hypermucoid strains. The isolates were screened for *mcr* genes using polymerase chain reaction. Whole-genome sequencing (WGS) and bioinformatics analysis were performed to characterize the genomes of PDR isolates. No plasmid-borne *mcr* genes were detected, and WGS analysis revealed that PDR isolates belonged to the high-risk clones: ST14 (n = 1), ST147 (n = 2), and ST383 (n = 2). They carried genes encoding extended-spectrum β-lactamases and carbapenemases, *bla*_CTX-M-15_ and *bla*_NDM-5_, on conjugative IncHI1B/IncFIB plasmids, illustrating the convergence of virulence and resistance genes. The most common mechanism of colistin resistance involved alterations in *mgrB*. Furthermore, deleterious amino acid substitutions were also detected within PhoQ, PmrC, CrrB, ArnB, and ArnT. Seven colistin-containing combinations were compared using the checkerboard experiment. Synergy was observed when combining colistin with tigecycline, doxycycline, levofloxacin, ciprofloxacin, sulfamethoxazole/trimethoprim, imipenem, or meropenem. The efficacy of colistin combined with either doxycycline or levofloxacin was assessed in vitro using a resistance modulation assay, and in vivo, using a murine infection model. In vitro, doxycycline and levofloxacin reversed colistin resistance in 80% and 73.3% of the population, respectively. In vivo, the colistin + doxycycline combination demonstrated superiority over colistin + levofloxacin, rescuing 80% of infected animals, and reducing bacterial bioburden in the liver and kidneys while preserving nearly intact lung histology.

**Conclusions:**

This study represents the first comparative in vitro and in vivo investigation of the efficacy of colistin + doxycycline and colistin + levofloxacin combinations in clinical PDR ColRKp isolates characterized at a genomic level.

**Supplementary Information:**

The online version contains supplementary material available at 10.1186/s13099-024-00667-z.

## Background

*Klebsiella pneumoniae*, a clinically problematic nosocomial bacterium, is one of the prevailing multidrug-resistant (MDR) pathogens that cause difficult-to-treat infections [1]. Over the past two decades, several studies have reported an increasing incidence of hospital-acquired carbapenem-resistant *K. pneumoniae* [2–5]*,* with an overall global prevalence of 28.69% [6]. Consequently, clinicians have been prompted to reconsider the use of colistin (COL) in clinical settings, deeming this polymyxin as the antibiotic of the twenty-first century [7, 8].

A cationic polypeptide, COL, was used from the 1950s until the mid-1970s. Afterward, its clinical use was discontinued due to nephrotoxicity and neurotoxicity concerns, alongside the parallel development of new antibiotics with better safety profiles [9]. However, COL returned in the mid-1990s with the emergence of extensively drug-resistant (XDR) pathogens, particularly carbapenem-resistant *K. pneumoniae* [9]. In the years that followed, the rise in COL utilization was regrettably mirrored by a surge in resistance rates to this polymyxin [10]. Resistance to COL is facilitated by genetic mutations at the chromosomal level and/or the horizontal transfer of mobile colistin resistance (*mcr*) genes. Both mechanisms ultimately trigger the remodeling of the molecular structure of lipopolysaccharide (LPS) through the addition of the cationic 4-amino-4-deoxy-l-arabinose (L-Ara4N) or phosphoethanolamine (pEtN) moieties. This process lowers the net negative charge of LPS and attenuates COL affinity for its LPS target [11, 12]. Among the chromosomal-mediated mechanisms, mutations in the regulatory systems of *phoPQ*, *pmrABC*, *mgrB*, and *crrB* are primarily associated with COL resistance in COL-resistant *K. pneumoniae* (ColRKp) [13]. Additionally, alterations in the chromosomal genes such as *pmrD,* the *arnBCADTEF* operon*,* and the global transcriptional regulator *ramA*, are thought to contribute to a secondary COL resistome [14]. Meanwhile, in the plasmid-encoded pathway, ten *mcr* alleles (*mcr-1* to *mcr-10*) have been identified in *Enterobacteriaceae* [15], with reports documenting the prevalence of clinical ColRKp strains carrying *mcr-1*, *mcr-3*, and *mcr-8* variants [16], and ColRKp of animal origin harboring *mcr-7* [17]. Resistance to COL significantly limits available treatment options and aggravates the risk of strains becoming resistant to all antibiotics, leading to the emergence of pandrug-resistant (PDR) isolates [18]. The global dissemination of Gram-negative PDR bacteria has been reported in 25 countries across five continents, with *K. pneumoniae* being responsible for approximately 24% of infections caused by PDR pathogens [19].

A promising solution for the management of these infections lies in the use of synergistic antibiotic combinations [19], which could serve as an alternative while awaiting the discovery and development of novel antimicrobials. Despite ample in vitro data evaluating such combinations, in vivo studies remain limited [18–22]. Clinical data guiding the treatment of these infections are insufficient and rely solely on a few case series and case reports in the literature [19]. Nevertheless, patients infected with Gram-negative PDR bacteria were the focus of a retrospective cohort study conducted at the University Hospital of Greece between 2010 and 2018 [23]. The study findings highlighted the effectiveness of COL-containing combinations as a promising empirical therapy, showing a 50% success rate among recipients and demonstrating superior efficacy compared to non-COL-based combinations [23]. In addition, previous in vitro studies have supported the role of COL-containing combinations against COL-resistant Gram-negative bacteria [18, 24], and research has demonstrated that these combinations significantly improve microbiological outcomes while potentially decreasing nephrotoxicity concerns associated with COL [25]. In this context, several investigations have assessed the synergistic activity of COL in the presence of different classes of antibiotics, including rifamycins, aminoglycosides, carbapenems, cephalosporins, fluoroquinolones, folate-pathway antagonists, tetracyclines, glycylcyclines, macrolides, and glycopeptides [18, 21, 22, 26].

The current study aims to shed light on the genomic characteristics of PDR ColRKp isolates, focusing on their resistomes, virulence attributes, and the different mechanisms conferring resistance to COL. To restore COL efficacy and lower its toxicity, we evaluate the effectiveness of COL-based combinations against these isolates. Within this scope, two antibiotics, doxycycline (DO) and levofloxacin (LE), were assessed for their ability to modulate COL resistance. To the best of our knowledge, we present the first experimental in vitro and in vivo comparative study investigating the efficacy of COL + DO and COL + LE combinations against clinical PDR ColRKp isolates. The data derived from the in vivo model are believed to provide crucial information for optimizing COL therapy and addressing pandrug resistance in ColRKp.

## Methods

### Bacterial isolates

*Klebsiella* spp. clinical isolates were periodically collected between January and July 2021 from the Alexandria University Diagnostic Medical Microbiology laboratory, affiliated with Alexandria Main University Hospital (AMUH), and from the laboratories of a private hospital in Alexandria. These isolates were obtained from clinical specimens of unidentified patients. The Ethics Committee at Alexandria University granted ethical approval, and the study results did not influence immediate patient care decisions. The isolates were obtained in terms of being COL-resistant *Klebsiella* spp. To verify their identity, the isolates were initially plated on MacConkey’s agar (HiMedia, Mumbai, India) to observe lactose fermentation. Subsequent steps included Gram staining and conventional, in-house prepared biochemical tests such as catalase, urease, triple-sugar iron agar, and IMVC (indole production, methyl red, Voges-Proskauer, and citrate utilization) tests. Further characterization to the species level was performed using the Vitek 2 compact system (bioMérieux, Marcy-L’Etoile, France). In total, 30 ColRKp isolates, numbered KP1 to KP30, were collected within 7 months.

### Antimicrobial resistance profile

The minimum inhibitory concentration (MIC) of COL (colistin sulfate, Pharmacure Pharmaceutical Industries, Cairo, Egypt) was assessed by the broth microdilution technique in triplicate using cation-adjusted Mueller–Hinton broth (CAMHB, HiMedia Laboratories, Mumbai, India) following the guidelines outlined in the Clinical Laboratory Standards Institute (CLSI, M100-ED31, 2021) [27] which set the breakpoint value for COL at 4 µg/mL. The susceptibility of the isolates to 18 antibiotics was tested using the Kirby-Bauer disk diffusion method. Antibiotic disks were procured from HiMedia Laboratories (Mumbai, India) for amoxicillin/clavulanate (AMC), piperacillin/tazobactam (P/T), ceftazidime (CAZ), ceftriaxone (CTR), cefepime (CPM), aztreonam (AT), imipenem (IPM), meropenem (MRP), ertapenem (ETP), gentamicin (GEN), amikacin (AK), azithromycin (AZ), doxycycline (DO), tigecycline (TGC), ciprofloxacin (CIP), and levofloxacin (LE). Disks for sulfamethoxazole/trimethoprim (SXT) and ceftazidime/avibactam (CZA) were obtained from Oxoid (Hampshire, UK). The testing was conducted using Mueller–Hinton agar (HiMedia Laboratories, Mumbai, India). Interpretation of the results was based on the breakpoints specified in CLSI, except for TGC, for which the breakpoint for *Enterobacteriaceae* defined by the Food and Drug Administration was followed [28].

### Phenotypic determination of virulence attributes

The investigation of phenotypic virulence attributes encompassed several techniques. First, the Anthony capsule staining technique [29] was employed to assess encapsulation. Next, the string test [30] was utilized to differentiate between hypermucoid (> 5 mm) and non-hypermucoid strains. Additionally, the crystal violet assay, conducted in triplicate, was employed to evaluate biofilm formation after 24 h using tryptone soya broth (HiMedia, Mumbai, India) supplemented with 2% glucose (Thermo Fisher Scientific, UK), as previously described [31]. *K. pneumoniae* ATCC 10031 was included as a reference positive control.

### Molecular detection of* mcr* genes

The isolates were screened for the presence of plasmid-borne *mcr* genes including *mcr-1*, *mcr-3*, *mcr-7*, and *mcr-8* utilizing conventional polymerase chain reaction (PCR). Primers were acquired from Macrogen (Seoul, South Korea); and their sequences, expected product sizes, and PCR annealing conditions are detailed in the Additional file: Table S1.

### Whole-genome sequencing (WGS) and bioinformatic analysis

The GeneJET Genomic DNA Purification Kit (Thermo Fisher Scientific, Vilnius, Lithuania) was employed to extract and purify the DNA of five PDR *K. pneumoniae* isolates (KP3, KP8, KP11, KP20, and KP22). WGS analysis was conducted at the Quadram Research Institute (Norwich, UK) on an Illumina NextSeq500 platform utilizing the Flowcell NextSeq 500/550 High Output Kit v2.5 (300 Cycles, Illumina Catalogue 20024908). Quality assessment of the generated FASTQ files was performed using fastp v0.23 [32], trimming poor-quality nucleotides with a Q-score < 20. Subsequently, the reads were de novo assembled into contigs using Shovill v1.1.0, and their quality was evaluated using QUAST v5.0.2 [33] and BUSCO v5.2.2 [34] for genome contiguity and completeness, respectively. Draft genomes underwent querying using tools hosted by the Center for Genomic Epidemiology (CGE) database for multilocus sequencing typing (MLST v2.0), detection of antimicrobial resistance (AMR) genes (ResFinder v4.1), and identification of chromosomal loci mutations (PointFinder v4.1) mediating resistance to cephalosporins, carbapenems, and fluoroquinolones. The *wzi* and *wzc* alleles, essential for capsule formation, along with heavy metal resistance genes, were identified by accessing the Institut Pasteur website. The *wzi* gene helps in capsule attachment to the bacterial surface, maintaining its stability, while the *wzc* gene regulates the production and export of the capsule, ensuring its proper formation. The Virulence Factor Database (VFDB) [35] and the Pasteur website were utilized to determine virulence genes. The capsular (K) and lipopolysaccharide (O-antigen) loci were typed using the Kaptive database. In silico investigation of chromosomal alterations associated with COL resistance was conducted using the BLASTn tool of the National Center of Biotechnology Information (NCBI), by comparing the investigated gene sequence in the draft genome to the wild-type gene sequence of *K. pneumoniae* subsp. *pneumoniae* HS11286 (GenBank accession number: NC_016845.1). If a mutation was identified at the genetic level, it was further analyzed using the tBLASTn tool to detect whether it conferred a change at the protein level. Subsequently, if a protein alteration was detected, its predicted effect, whether neutral or deleterious, was determined using the Protein Variation Effect Analyzer tool (PROVEAN). The CSIPhylogeny server [36] was utilized to infer the phylogenetic relatedness of the tested isolates, using NC_009648.1 as the reference genome. The resulting phylogenetic tree was then visualized using Interactive Tree of Life v6.5 (iTOL) [37].

### Construction and comparison of IncHI1B/IncFIB plasmids

Plasmid sequences were reconstructed and typed from WGS assemblies using MOB-suite software V3.0.0 [38]. The plasmid-labeled contigs were queried on the Pasteur website to detect virulence and heavy metal resistance genes. The five plasmids of IncHI1B/IncFIB replicon types were reference-mapped against their nearest neighbor plasmid (accession number CP137372) using the BLASTn tool to generate the complete plasmid sequences. The NCBI Prokaryotic Genome Annotation Pipeline (PGAP) tool was used for the annotation of plasmids [39]. The circular comparison map, illustrating the chosen plasmids was created using the CGview server v1.1.2.

### Antimicrobials for checkerboard assay

TGC (Tegasterk^®^, 50 mg lyophilized powder for IV infusion), CIP (Ciprocin^®^, 10 mg/mL for IV infusion), LE (Tavanic®, 5 mg/mL solution for IV infusion), IPM (Tienam^®^, 500 mg IV vial), and MRP (Meronem^®^, 1000 mg IV vial) were purchased from pharmacy stores. Sulfamethoxazole (report number: QC19110320 and batch number: A20211910039-0300), trimethoprim (report number: QC19090557 and batch number: A50111908016-0500), and DO (report number: QC2101887 and batch number: A201701100) powders were kindly provided by Pharco Pharmaceuticals Company, Egypt.

### Checkerboard assay

Seven COL-containing combinations (COL + TGC, COL + DO, COL + CIP, COL + LE, COL + SXT, COL + IPM, and COL + MRP) were tested in twofold serial dilutions against the five PDR isolates from the ColRKp collection. The concentration of COL in these combinations ranged from 4 X MIC to 1/256 X MIC, while the antibiotics were applied at concentrations ranging from 4 X MIC to 1/16 X MIC, following the previously described protocol [40]. The MIC of each antibiotic was determined according to the CLSI 2021 recommendations [27], both alone and in combination, to evaluate the drug-drug combinatorial effect through the calculation of the fractional inhibitory concentration index (ΣFICI) using the following equation:

$$\sum {{\text{FICI}}} = {{{\text{MIC}}_{{\text{drug A in combination}}} } \mathord{\left/ {\vphantom {{{\text{MIC}}_{{\text{drug A in combination}}} } {{\text{MIC}}_{{\text{drug A alone}}} }}} \right. \kern-0pt} {{\text{MIC}}_{{\text{drug A alone}}} }} + {{{\text{MIC}}_{{\text{drug B in combination}}} } \mathord{\left/ {\vphantom {{{\text{MIC}}_{{\text{drug B in combination}}} } {{\text{MIC}}_{{\text{drug B alone}}} .}}} \right. \kern-0pt} {{\text{MIC}}_{{\text{drug B alone}}} .}}$$

ΣFICI results were interpreted as follows: synergism when ΣFICI ≤ 0.50, additive effect when ΣFICI > 0.50 and < 1, indifference when ΣFICI ≥ 1 and < 2, and antagonism when ΣFICI ≥ 2 [26]. Additionally, the MIC decrease factor (MDF) for the tested isolates was estimated using the following equation: MDF = MIC _antibiotic alone_ / MIC _antibiotic in combination_ [41].

### Resistance modulation assay

We further tested the COL + DO and COL + LE combinations against the entire ColRKp collection to detect the COL resistance-modifying effect of DO and LE at subinhibitory concentrations using the resistance modulation assay [42]. Initially, the MICs of DO and LE were determined against all isolates using the broth microdilution technique as indicated by the CLSI 2021 guidelines [27]. Subsequently, a sub-inhibitory concentration (0.25 X MIC) of each DO and LE was tested as a resistance modulator when combined with COL, which was used at concentrations ranging from 256 to 0.25 µg/mL. The modulation factor (MF), representing the fold reduction in the MIC of COL, was calculated using the following equation: MIC _COL alone_/MIC _COL in combination_ [42]. The MF was employed to elucidate the resistance-modifying effects of DO and LE.

### In vivo infection model

Six-week-old female Swiss albino mice weighing 20 ± 2 g were housed in the Faculty of Pharmacy, Alexandria University animal facility, where a constant temperature of 23 ± 2 °C was maintained with 30–70% relative humidity. Ad libitum access to standard food and water was provided to the mice. All animal experiments were conducted in accordance with international and institutional ethical guidelines following approval from the Institutional Animal Care and Use Committee (IACUC) of the Faculty of Pharmacy, Alexandria University, Egypt. A murine infection model was established as previously described by Mohamed et al. [43] with some modifications. Mice were intraperitoneally (i.p.) inoculated with 200 µL containing 10 X minimal lethal dose (1.7 × 10^8^ CFU/mouse) of KP20 isolate combined with porcine mucin (Sigma Aldrich, USA) at a final concentration of 5%. This inoculum was sufficient to induce mortality in 100% of untreated mice within 24 h post-infection.

### Survival experiment

The KP20-infected mice were allowed to develop pandrug resistance-associated bacteremia for 2 h. At 2 h post-infection, the mice were divided into 6 groups of 10 mice each and administrated an initial dose of the following treatments i.p.: saline (control), COL (colistin sulfate, Pharmacure Pharmaceutical Industries, Cairo, Egypt) used at dose 5 mg/kg/day [44], DO (doxycycline hydrochloride, Pharco Pharmaceutical Industries, Cairo, Egypt) used at dose 50 mg/kg/day [45], LE (Tavanic^®^, 5 mg/mL solution for IV infusion) used at dose 150 mg/kg/day [46], COL + DO (5 mg/kg/day + 50 mg/kg/day), and COL + LE (5 mg/kg/day + 150 mg/kg/day). A second dose was given 24 h after the initial dose. The animals were monitored for survival over 15 days to evaluate the in vivo efficacy of the administrated treatment regimen.

### Organ bioburden and histopathological examination

The aforementioned murine bacteremia model was applied to a total of 36 mice divided into control (receiving saline) and treatment groups (receiving one of the following: COL, DO, LE, COL + DO, and COL + LE). Each group consisted of 6 mice, with each mouse receiving a single dose 2 h post-infection. The bacterial burden in the liver and kidneys was determined after 24 h or immediately after the animal's death. At the end of the experiment, the surviving mice were euthanized by cervical dislocation, followed by aseptic dissection to collect and homogenize the organs in sterile saline. A tenfold serial dilution was performed on the homogenates, which were then plated on MacConkey’s agar. The viable bacterial count (log CFU/organ) was determined after incubation at 37 °C for 24 h. Based on the results of the survival experiment, lung samples were obtained for the histopathological examination from the control, COL, DO, and COL + DO groups, in addition to a negative control group where mice were not challenged with KP20 isolate and received 200 µL of 0.9% saline instead. The lungs of each mouse were dissected and preserved in 10% buffered formalin (EL Nasr Pharmaceutical Chemicals Company, Egypt). Three 5-mm-thick serial sections were cut from paraffin-embedded tissue and stained with hematoxylin and eosin (H&E). The degree of inflammation over the total area of each serial section was examined and graded by a histopathologist blinded to the sample origin, according to previously adopted methods [47, 48]. The assessed inflammatory parameters included vascular congestion (graded 0 to 4), alveolar destruction (0–4), perivascular edema (0–3), and interstitial inflammation (0–3). A total inflammatory score (0–14) was calculated as the sum of the individual scores reported. Both negative (uninfected) and positive (infected but untreated) lung controls were used for comparative purposes.

### Statistical analysis

Kaplan Meier survival analysis and the log-rank (Mantel-Cox) test were performed to analyze the mice survival experiment using GraphPad Prism v8.0.2 (GraphPad Software, San Diego, CA, USA). Bacterial count comparisons between the different mice groups were evaluated using one-way ANOVA with Tukey’s multiple comparisons test followed by post hoc analysis. Histopathology data were analyzed using the Kruskal–Wallis followed by Dunn’s multiple comparison tests.

## Results

### Bacterial isolates and clinical characteristics

Based on the electronic records from the laboratories where the isolates were collected, the isolation rate of *Klebsiella* species was approximately 400–500 isolates per month. Over 7 months, a total of 30 COL-resistant *Klebsiella* spp. clinical isolates were gathered. Among these, 24 were obtained from the laboratories of a private hospital, while the remaining 6 isolates were sourced from AMUH. The isolates were initially identified using standard phenotypic techniques and further characterized using the Vitek 2 compact system, which confirmed their identity as *K. pneumoniae* subspecies *pneumoniae.* A significant proportion of the isolates (83.3%) were recovered from adult and neonatal intensive care units (ICUs), whereas 16.67% were retrieved from hospitalized patients in different hospital wards. The isolates were reclaimed from various clinical specimens, including blood (n = 11), mini-bronchoalveolar lavage (n = 6), urine (n = 5), wound (n = 3), sputum (n = 2), central venous catheter (n = 1), and pus (n = 1). The study collection included multiple age groups: neonates (n = 5, 3–20 days), children (n = 1, 8 years), adolescents (n = 2, 13 and 18 years), adults (n = 10, 23–64 years), and geriatrics (n = 8, 65–92 years). The clinical origin of the isolates and the demographic data, including the date of collection, source, hospital unit, gender, and age, are provided in the Additional file: Table S2.

### Antibiotic susceptibility testing

**Fig. 1:**
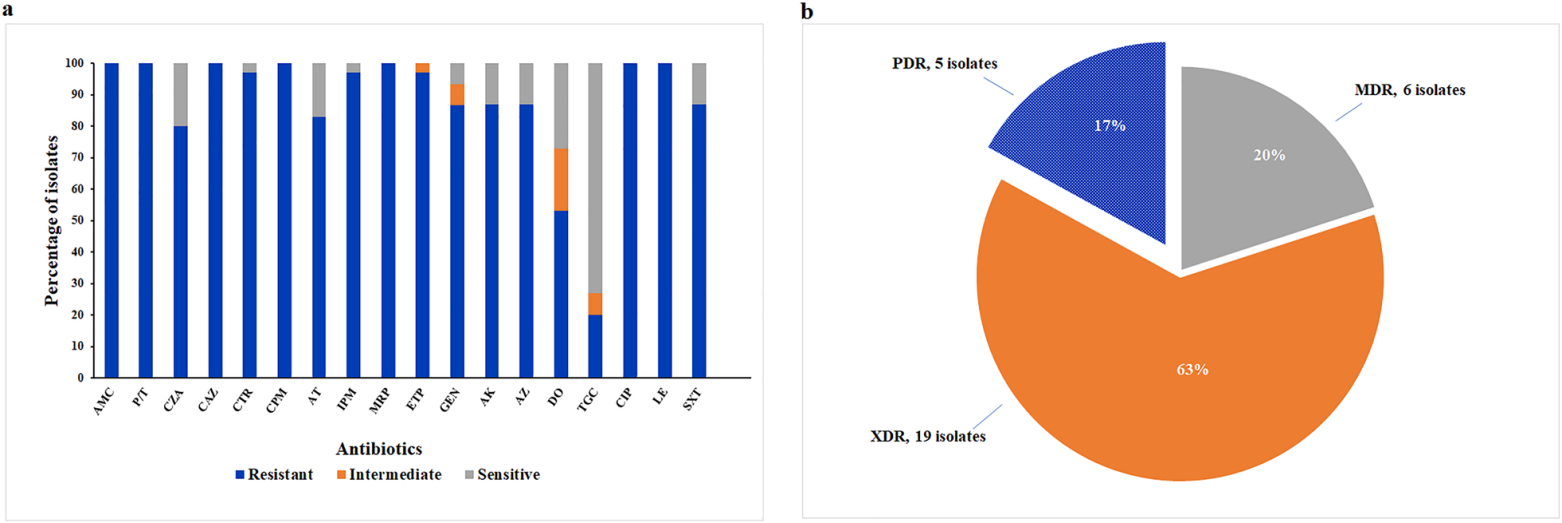
**a** Percentages of sensitivity, intermediate susceptibility, and resistance to the tested antibiotics among 30 colistin-resistant *K. pneumoniae* isolates. *AMC* stands for amoxicillin/clavulanate, *P/T* piperacillin/tazobactam, *CZA* ceftazidime/avibactam, *CAZ* ceftazidime, *CTR* ceftriaxone, *CPM* cefepime, *AT* aztreonam, *IPM* imipenem, *MRP* meropenem, *ETP* ertapenem, *GEN* gentamicin, *AK* amikacin, *AZ* azithromycin, *DO* doxycycline, *TGC* tigecycline, *CIP* ciprofloxacin, *LE* levofloxacin, and *SXT* sulfamethoxazole/trimethoprim; **b** Prevalence of multidrug-resistant (MDR), extensively drug-resistant (XDR), and pandrug-resistant (PDR) isolates among the studied collection

**Fig. 2:**
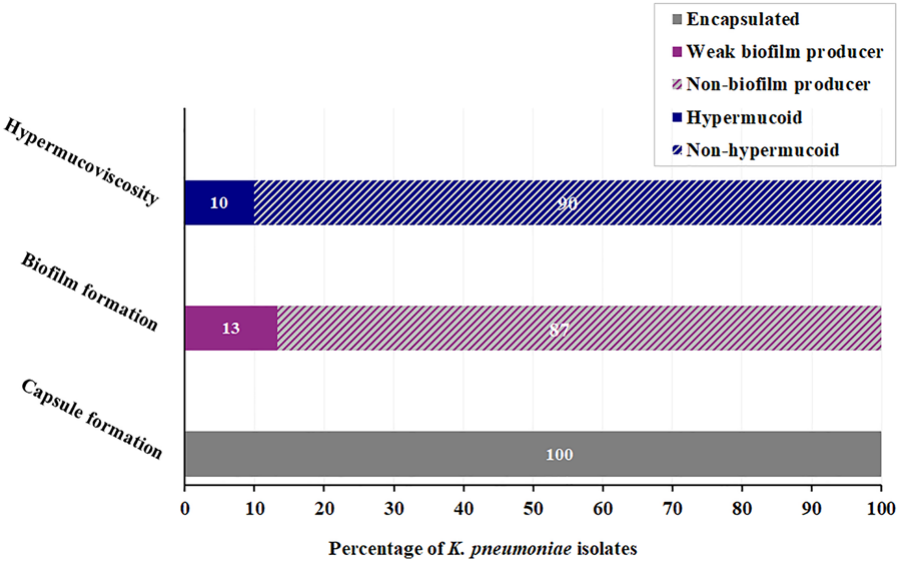
Prevalence of different virulence factors in the tested colistin-resistant *K. pneumoniae* isolates

All collected isolates were confirmed to be COL-resistant, with MICs ranging from 4 to 512 µg/mL. The MIC_50_ and MIC_90_ values were 32 and 64 µg/mL, respectively (Additional file: Table S3). The selection of the 18 antibiotics used in the Kirby–Bauer disk diffusion technique was guided by the CLSI 2021 recommendations for *K. pneumoniae* infections. All ColRKp isolates were resistant to AMC, P/T, CAZ, CPM, MRP, ETP, CIP, and LE. Increasing levels of non-susceptibility, ranging from 73 to 97%, were observed for DO, CZA, AT, AK, AZ, SXT, GEN, CTR, and IPM (Fig. 1a). While most of the isolates exhibited concerning levels of resistance to 17 out of 18 of the tested antibiotics, a sensitivity level of 73% to TGC was observed among the studied collection (Fig. 1a). Based on the obtained antibiogram, the resistance status of each isolate was determined (Additional file: Table S3), and the isolates were categorized to MDR, extensively drug-resistant (XDR), and PDR (Fig. 2b). Six isolates were classified as MDR by being resistant to a minimum of one antibiotic in three or more of the tested antimicrobial categories. Nineteen isolates were classified as XDR, showing non-susceptibility to at least one agent in the investigated antimicrobial classes except one or two. Seventeen percent of the population (KP3, KP8, KP11, KP20, and KP22 isolates) exhibited a PDR phenotype, being resistant to all tested antibiotics.

### Virulence profile

Virulence profiling was determined by assessing the isolates’ encapsulation, biofilm-forming ability, and hypermucoidy (Fig. 2). Using the Anthony capsule staining technique, all tested isolates showed clear halo-like structures surrounding the stained bacterial cells, confirming the prevalence of encapsulated strains within the population. The crystal violet assay segregated the ColRKp isolates into weak biofilm formers, including KP4, KP11, KP18, and KP23, and non-biofilm formers (87%, n = 26). Ten percent of the isolates (KP3, KP18, and KP20) showed a positive string test result for hypermucoidy, generating a viscous string of > 5 mm when a bacterial colony was touched with a bacteriological loop. The remaining strains were non-hypermucoid (Fig. 2).

### Genomic characterization of PDR strains and genetic determinants of pandrug resistance

**Fig. 3:**
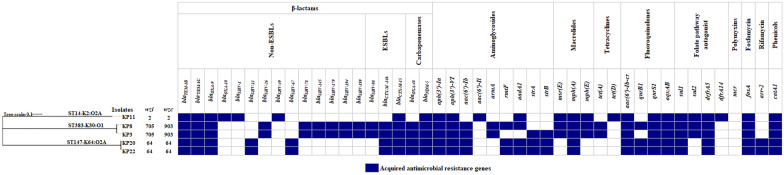
Heatmap depicting the antimicrobial resistance genes of the five pandrug-resistant *K. pneumoniae* isolates. The resistance profile is mapped as gene present (colored) or absent (white). On the left, a rooted phylogenetic tree is visualized using iTOL tool displaying isolates of the same sequence types (STs) and capsular polysaccharides (K-types) clustered together. STs, K- and O-types are indicated above each well-defined branch. *wzi* and *wzc* alleles for each isolate are shown

The five PDR isolates (KP3, KP8, KP11, KP20, and KP22) were subjected to genomic analysis using WGS. The de novo-assembled genomes ranged in size from 5.7 to 6 Mb, which is typical of the *K. pneumoniae* genome. On average, 310 contigs constituted the draft genome with a median N50 value of 109,720 bp and a mean G + C content of 56.60% (Additional file: Table S4). The genome sequences were used to construct a phylogenetic tree to elucidate the evolutionary relationships among the isolates (Fig. 3). The tree comprised three main branches representing the ancestry lineages to which the isolates belong, including the sequence types (ST): ST14 (n = 1), ST147 (n = 2), and ST383 (n = 2). Capsular diversity was noted among the strains, evidenced by the various detected alleles of *wzc* and *wzi* genes responsible for capsule biosynthesis. K- and O-typing revealed the presence of the following capsular polysaccharides: K2 (n = 1), K30 (n = 2), and K64 (n = 2), along with O1 (n = 2) and O2A (n = 3) antigens among the PDR strains (Fig. 3).

Resistance profiles of the sequenced isolates were investigated using ResFinder and PointFinder, revealing a myriad of AMR genes (Fig. 3) and chromosomal loci mutations (Additional file: Table S5), consistent with the displayed PDR phenotype. All isolates were extended-spectrum β-lactamase (ESBL) and carbapenemase producers harboring *bla*_CTX-M-15_ and *bla*_NDM-5_. Additional ESBL-encoding genes included *bla*_CTX-M-14b_ (n = 4) and *bla*_SHV-98_ (n = 2). The *bla*_OXA-48_ carbapenemase was detected in 4 out of 5 isolates, whereas *bla*_NDM-1_, *bla*_KPC_, *bla*_IMP_, and *bla*_VIM_ were not found among the studied isolates. Other β-lactamases included non-ESBLs such as *bla*_TEM-1B_, *bla*_TEM-1C_, *bla*_OXA-9_, *bla*_OXA-10_, and different *bla*_SHV_ variants, with at least five non-ESBL-encoding genes detected in each isolate. Multiple determinants for aminoglycoside resistance were identified, including *armA* and *rmtF*, which are responsible for pan-aminoglycoside resistance, and were present in 40% and 60% of the PDR isolates, respectively. The aminoglycoside resistance genes *aph(3*′*)-Ia*, *aph(3′)-VI*, and *aac(6′)-Ib-cr* were found in all sequenced genomes, with the latter gene also mediating resistance to CIP. Furthermore, three pathways conferring resistance to fluoroquinolones were detected, including chromosomal mutations within the *parC*, *gyrA*, and *acrR* genes (Additional file: Table S5); the chromosomally mediated efflux pump, *oqxAB*; and the plasmid-borne genes, *qnrB1* and *qnrS1*. Notably, mutations in the *acrR* gene, the repressor of the AcrAB multidrug efflux pump, not only contribute to fluoroquinolones resistance but also confer multidrug resistance to chloramphenicol, trimethoprim, macrolides, and tetracyclines [49]. The acquisition of *tet(A)* and *tet(D)* genes accounted for DO resistance in isolates of ST383 and ST14, respectively. Distinct variants of *sul* and *dfrA* genes conveying resistance to SXT co-existed among the PDR isolates, with a distinct prevalence of *sul1* and *dfrA5*. Additional resistance determinants were detected for other classes of antibiotics, including chloramphenicol acetyltransferases, *catA1,* driving resistance to chloramphenicol; *msr(E)*, *mph(A)*, and *mph(E)* encoding resistance to macrolides; *fosA* coding for fosfomycin resistance; and *arr-2*, mediating resistance to rifamycin.

The genetic determinants of virulence were found to be localized across the chromosomes and plasmids of different replicon types identified in the sequenced isolates. Each isolate possessed core pathogenicity elements on its chromosome, including the *fim* and *mrk* operons which code for Type 1 and Type 3 fimbriae, respectively. All PDR isolates harbored the core siderophore system enterobactin (*entABCDEFS*) along with aerobactin (*iucABCD* and *iutA*) and a partially complete salmochelin system consisting of *iroE* and *iroN* genes. Yersiniabactin, another siderophore system encoded by *ybtAEPQSTUX*, *irp1/2*, and *fyuA*, was detected in 3 out of 5 isolates belonging to ST147 and ST383. An alternative iron acquisition pathway, *kfu* (*Klebsiella* ferric uptake), was exclusively detected in the KP11 chromosome. The hypervirulence biomarker *rmpA* (regulator of mucoid phenotype A) was identified in KP3, which exhibited phenotypic expression of hypermucoidy but was absent in the hypermucoid KP20 isolate (Table 1). The genes responsible for allantoin utilization, *allS* and *allABCDR*, were not detected in any PDR strains.

**Table 1:** Localization of virulence and heavy metal resistance genes across chromosomes and plasmids of different replicon types and mobilities in the pandrug-resistant *K. pneumoniae* isolates

Isolate code	Chromosome/plasmid	Replicon types	Predicted mobility	Virulence genes	Heavy metal resistance genes
KP3	Chromosome	–		*mrkABCDFHIJ*, *fimABCDEFGHIK entABCDEFS*, *fyuA*, *irp1*, *ybtAEPQSTUX*, *iroE*,* iroN*	*silR*
	pEGY_KP3^a^	IncHI1B/IncFIB	Conjugative	*iutA*, *iucABCD*,* rmpA*	*terWXY*
	Plasmid KP3-1	IncL/M	Conjugative	–	–
	Plasmid KP3-2	ColRNAI	Non-mobilizable	–	–
	Plasmid KP3-3	ColRNAI	Mobilizable	–	–
KP8	Chromosome	–	–	*mrkABCDFHIJ*, *fimABCDEFGHIK*, *entABCDEFS*, *iroE*,* iroN*	*silR*
	pEGY_KP8^a^	IncHI1B/IncFIB	Conjugative	*iutA*,* iucABCD*	*terABCDEWXYZ*
	Plasmid KP8-1	IncL/M	Conjugative	–	–
	Plasmid KP8-2	IncFIB/IncFII_K_	Conjugative	–	–
	Plasmid KP8-3	ColRNAI	Non-mobilizable	–	–
	Plasmid KP8-4	ColRNAI	Mobilizable	–	–
KP11	Chromosome	–	–	*mrkABCDFHIJ*, *fimABCDEFGHIK*, *entABCDEFS*, *iroE*, *iroN*, *iutA*,* kfuAB*	*silR*
	pEGY_KP11^a^	IncHI1B/IncFIB	Conjugative	*iucABCD*,* iutA*	*terABCDEWXYZ*
	Plasmid KP11-1	IncFIB	Non-mobilizable	–	–
	Plasmid KP11-2	IncFIB_K_/IncFII_K_	Conjugative	–	*arsABCDR, merCPRT, pcoABCDERS, silABCEFGPRS*
	Plasmid KP11-3	ColRNAI	Mobilizable	–	–
KP20	Chromosome	–	–	*mrkABCDFHIJ*, *fimABCDEFGHIK*, *entABCDEFS*, *fyuA*, *irp1*, *irp2*, *ybtAEPQSTUX*, *iroE*, *iroN*,* iutA*	*silA, silR*
	pEGY_KP20^a^	IncHI1B/IncFIB	Conjugative	*iucABCD*,* iutA*	*terABCDEWXYZ*
	Plasmid KP20-1	IncL/M	Conjugative	–	–
	Plasmid KP20-2	IncFIB/IncFII_K_	Conjugative	–	–
	Plasmid KP20-3	IncFIB	Non-mobilizable	–	–
	Plasmid KP20-4	ColRNAI	Non-mobilizable	–	–
KP22	Chromosome	–	–	*mrkABCDFHIJ*, *fimABCDEFGHIK*, *entABCDEFS fyuA*, *irp1*, *irp2*, *ybtAEPQSTUX*, *iroE*, *iroN*,* iutA*	*silA, silR*
	pEGY_KP22^a^	IncHI1B/IncFIB	Conjugative	*iucABCD*,* iutA*	*terABCDEWXYZ*
	Plasmid KP22-1	IncL/M	Conjugative	–	–
	Plasmid KP22-2	IncFIB/IncFII_K_	Conjugative	–	–
	Plasmid KP22-3	ColRNAI	Non-mobilizable	–	–
	Plasmid KP22-4	IncFIB	Non-mobilizable	–	–
	Plasmid KP22-5	Col(BS512)	Non-mobilizable	–	–

^a ^Plasmids were submitted to the National Center and Biotechnology Information Institute

Several heavy metal resistance genes were identified using the Pasteur website. All isolates harbored tellurium (*ter*) and silver (*sil*) resistance genes, encoded either on their chromosomes or carried on their IncHI1B/IncFIB plasmids. Meanwhile, mercury (*mer*), arsenic (*ars*), and copper (*pco*) resistance genes were exclusively found on the KP11-2 plasmid (Table 1).

### Characterization of IncHI1B/IncFIB plasmids

**Fig. 4:**
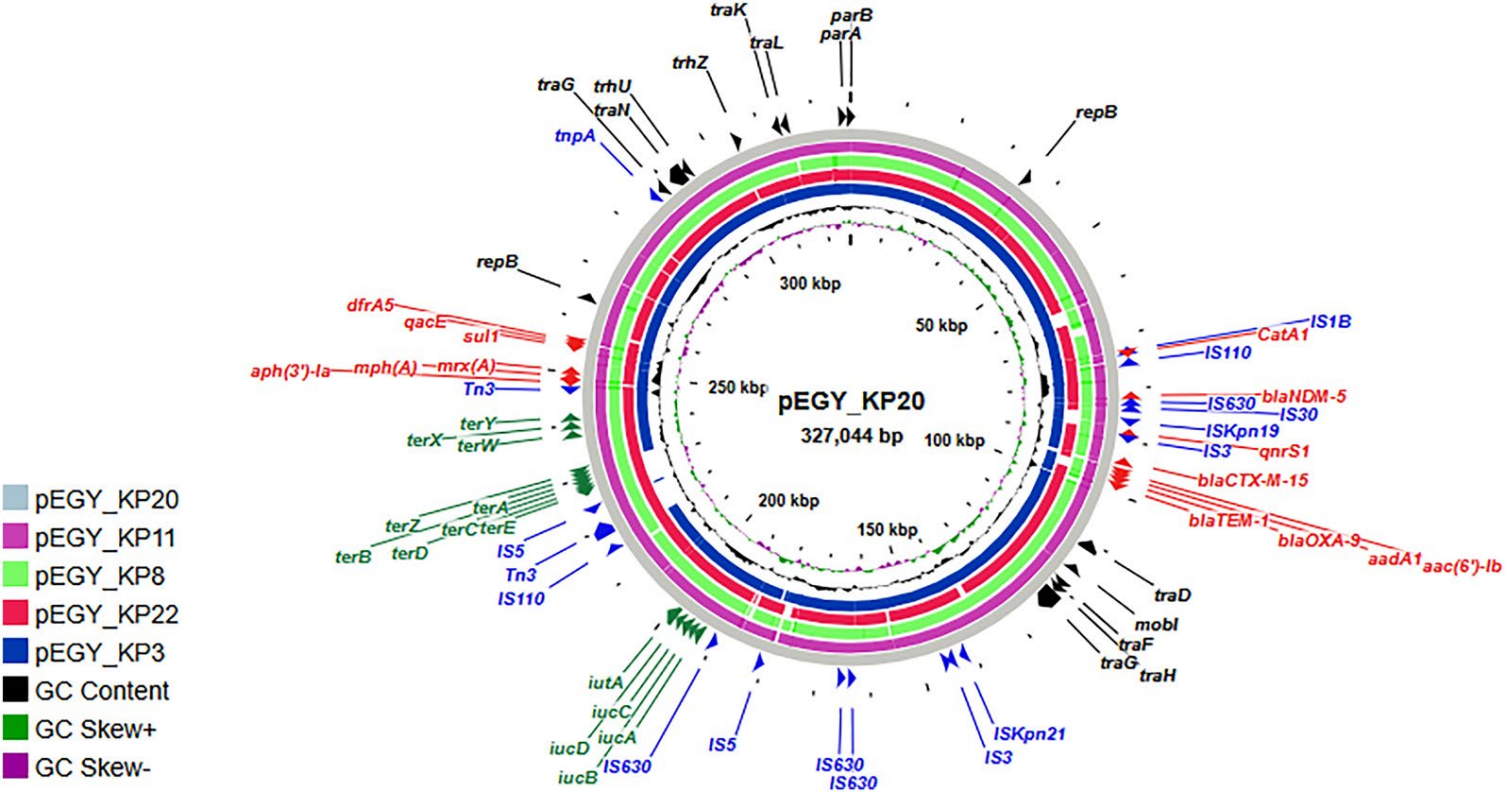
CGview comparison map of pEGY_KP20 plasmid with similar conjugative IncHI1B/IncFIB plasmids harbored by the pandrug-resistant *K. pneumoniae* isolated in this study. The backbone of the map represents the coding regions of pEGY_KP20 (grey) with its size indicated in the center. The labels at the outer ring denote the annotation of genes related to virulence and heavy metal resistance (green), antibiotic resistance (red), and transposases (blue). Genes responsible for plasmid replication, conjugation, and segregation are shown in black. Genomic regions identified by BLASTn are displayed in solid color, while white gaps indicate areas not covered by BLASTn

Given their significance as the only plasmids encoding virulence and heavy metal resistance genes, the complete sequences of the conjugative IncHI1B/IncFIB plasmids carried by the five PDR isolates were generated and deposited in NCBI (GenBank accessions CP154635 to CP154639). Comparative analysis of pEGY_KP20 against pEGY_KP3, pEGY_KP8, pEGY_KP11, and pEGY_KP22 plasmids using BLASTn revealed high sequence similarity, reaching 99.88%, 99.89%, 99.92%, and 99.98%, with query coverage of 94%, 96%, 99%, and 94%, respectively. The plasmid designated pEGY_KP20 was chosen as the referral plasmid since it was carried by the isolate further selected for in vivo modeling. pEGY_KP20 was found to be a large (~ 327 kbp) mosaic plasmid created by the fusion of two different backbones, IncHI1B and IncFIB. This plasmid distinctly illustrated convergence with a simultaneous carriage of virulence and AMR determinants. It encoded the aerobactin siderophore system (*iucABCD* and *iutA*), the tellurium resistance genes (*terABCDEWXYZ*), and was heavily shaped by an array of AMR genes conferring resistance to aminoglycosides, β-lactams, carbapenems, macrolides, fluoroquinolones, folate pathway antagonists, and phenicols. The mosaic architecture of the pEGY_KP20 plasmid unveiled the distribution of multiple insertion sequences, transposases, and genes necessary for plasmid replication (*rep*), conjugation (*mobI*, *tra*, and *trh*), and maintenance (*par*) (Fig. 4).

### Molecular mechanisms of colistin resistance

PCR screening for *mcr-1, mcr-3, mcr-7,* and *mcr-8* variants indicated the absence of these plasmid-borne COL resistance genes among all 30 isolates. This was further confirmed by querying the assembled contigs on ResFinder in the PDR strains. To unveil the molecular mechanisms mediating resistance to COL, chromosomal alterations in *phoPQ*, *pmrABCD*, *crrB, arnBCADTEF* operon, *ramA, mgrB*, and its promoter, along with the surrounding genetic environment including *kdgR*, *yobH*, *yebO*, *yobF*, and *cspC* were inspected. The tBLASTn tool detected amino acid substitutions within PhoQ (E397G), PmrC (S257L), CrrB (A200Y), ArnB (G47D), and ArnT (L54H). These substitutions were predicted to be deleterious on the protein level by the PROVEAN tool. Alterations in *mgrB* were the most common mechanism conferring resistance to COL, encountered in 60% of the PDR isolates (Table 2). The KP3 isolate had a chromosomal mutation of guanine into adenine at position 109 (G109A), generating an unfunctional MgrB with serine instead of glycine at position 37 (G37S). Splitting of *mgrB* was encountered in the KP11 isolate through insertional inactivation by a member of the IS1 family, IS*Kpn14*, at nucleotide position 35. Additionally, mutation of cytosine to thymine at nucleotide position 88 (C88T) of *mgrB* led to a premature stop codon, generating an incomplete MgrB with 29 amino acids instead of the expected 47 in the KP22 isolate. Conversely, the *mgrB* promoter and the surrounding genetic environment of the transcriptional regulator KdgR, YobH, YebO, YobF, and the cold shock protein CspC, did not reveal any genetic modifications upon analysis. Similarly, no substitutions were detected in PhoP, RamA, ArnC, and ArnE, while neutral amino acid substitutions were identified in PmrABCD, CrrB, ArnB, ArnA, ArnD, ArnT, and ArnF (Table 2). Collectively, these findings highlight diverse chromosomal alterations as the main mechanism underlying COL resistance among PDR *K. pneumoniae* isolates.

**Table 2:** Colistin MIC and the genetic alterations associated with colistin resistance in pandrug-resistant *K. pneumoniae* isolates

Isolate code	COL^a^ MIC (µg/mL)	PhoQ	PmrA	PmrB	PmrC	PmrD	CrrB	*mgrB /* MgrB	ArnB	ArnA	ArnD	ArnT	ArnF	Others^c^
KP11	128	WT^b^	WT	A223T G233R	F27C **S257L** R319Q	WT	Absent	**IS*Kpn14* at position 35**	A112D I126V **G47D**	WT	S164P	WT	WT	WT
KP8	8	**E397G**	N131D G144D D149E N219H	N82S M152V A205T G233R	F27C L39V R319Q	T60M	Absent	WT/WT	A112D D217N D285E	WT	WT	I117V	F48V	WT
KP3	16	WT	N131D G144D D149E N219H	N82S M152V A205T G233R	F27C L39V R319Q	T60M	Absent	G109A/**G37S**	A112D D217N D285E	WT	WT	**L54H** I117V	F48V	WT
KP20	512	WT	WT	WT	V50L A135P R319Q	WT	V194_S195insSS^d^ S196G S197G G198A **A200Y**	WT / WT	WT	S18A T185A	WT	L114M I117V H156Q	WT	WT
KP22	16	WT	WT	WT	V50L A135P R319Q	WT	WT	C88T/Q30*	WT	S18A T185A	WT	L114M I117V H156Q	WT	WT

^a^* COL *colistin, ^b^* WT* wild-type, ^c^ Others refer to PhoP, Kdgr, YobH, YobF, YebO, CspC, RamA, ArnC, and ArnE. ^d ^V194_S195insSS indicates the insertion of two serine amino acids between valine at position 194 and serine at position 195. *Represents a stop codon. The bold format indicates deleterious amino acid substitution predicted by PROVEAN and the genetic alterations associated with colistin resistance

### Assessment of colistin-containing combinations

Seven COL-containing combinations were evaluated by checkerboard assay to tackle the challenge of pandrug resistance. The COL + DO and COL + LE combinations were prioritized for consistently showing synergy against all five tested PDR isolates and for dropping the MIC of COL to the susceptibility breakpoint (2 µg/mL) when DO and LE were used at concentrations ranging from 2 to 32 µg/mL. Combinations including COL + TGC, COL + CIP, and COL + SXT demonstrated synergy against 4/5 of the tested isolates and an additive effect against a single isolate. However, the combinatory effects of COL + IPM and COL + MRP ranged from indifference, additivity, to synergy (Table 3). Accordingly, compared to the other tested combinations, the carbapenem-containing ones were considered the least successful. The MDF of COL and those of the combined antibiotics ranged from 2 to 256 folds (Table 3).

**Table 3:** Combined activity of colistin with selected antibiotics against pandrug-resistant *K. pneumoniae* isolates

MIC (µg/mL)/MDF^a^/ΣFICI^b^/Outcome^c^	KP3	KP8	KP11	KP20	KP22
MIC of COL	16	8	128	512	16
MIC of TGC	8	8	8	8	4
MICs of COL + TGC MDF (ΣFICI/Outcome)	4 + 0.5 4 + 16 (0.31/syn)	4 + 0.125 2 + 64 (0.52/add)	16 + 2 8 + 4 (0.38/syn)	64 + 2 8 + 4 (0.38/syn)	2 + 1 8 + 4 (0.38/syn)
MIC of DO	32	32	128	32	8
MICs of COL + DO MDF (ΣFICI/Outcome)	2 + 8 8 + 4 (0.38/syn)	2 + 4 4 + 8 (0.38/syn)	2 + 32 64 + 4 (0.27/syn)	2 + 8 256 + 4 (0.25/syn)	2 + 2 8 + 4 (0.38/syn)
MIC of CIP	256	256	8	256	256
MICs of COL + CIP MDF (ΣFICI/Outcome)	2 + 4 8 + 64 (0.14/syn)	4 + 4 2 + 64 (0.52/add)	4 + 2 32 + 4 (0.29/syn)	16 + 32 32 + 8 (0.17/syn)	2 + 32 8 + 8 (0.25/syn)
MIC of LE	64	128	8	128	128
MICs of COL + LE MDF (ΣFICI/Outcome)	2 + 8 8 + 8 (0.25/syn)	2 + 32 4 + 4 (0.50/syn)	2 + 2 64 + 4 (0.27/syn)	4 + 32 128 + 4 (0.26/syn)	2 + 16 8 + 8 (0.25/syn)
MIC of IPM	32	32	64	16	32
MICs of COL + IPM MDF (ΣFICI/Outcome)	8 + 1 2 + 32 (0.53/add)	4 + 16 2 + 2 (1/ind)	8 + 4 16 + 16 (0.13/syn)	32 + 2 16 + 8 (0.19/syn)	8 + 2 2 + 16 (0.56/add)
MIC of MRP	64	128	128	128	128
MICs of COL + MRP MDF (ΣFICI/Outcome)	8 + 1 2 + 64 (0.52/add)	4 + 64 2 + 2 (1/ind)	8 + 8 16 + 16 (0.13/syn)	64 + 4 8 + 32 (0.16/syn)	4 + 32 4 + 4 (0.50/syn)
MIC of SXT	1024/19456	1024/19456	1024/19456	512/9728	512/9728
MICs of COL + SXT MDF (ΣFICI/Outcome)	2 + 64/1216 8 + 16/16 (0.25/syn)	1 + 128/2432 8 + 8/8 (0.38/syn)	32 + 32/608 4 + 32/32 (0.31/syn)	256 + 64/1216 2 + 8/8 (0.75/add)	4 + 64/1216 4 + 8/8 (0.50/syn)

^a ^*MDF* MIC decrease factor; ^b ^*ΣFICI* Fractional inhibitory concentration index, ^c ^Outcome: *syn* refers to synergism, *add* to additive effect, and *ind* to indifference.* COL* refers to colistin,* TGC* tigecycline,* DO* doxycycline,* CIP* ciprofloxacin,* LE* levofloxacin,* IPM* imipenem,* MRP* meropenem, and* SXT* sulfamethoxazole/trimethoprim

### Resistance modulation assay

**Fig. 5:**
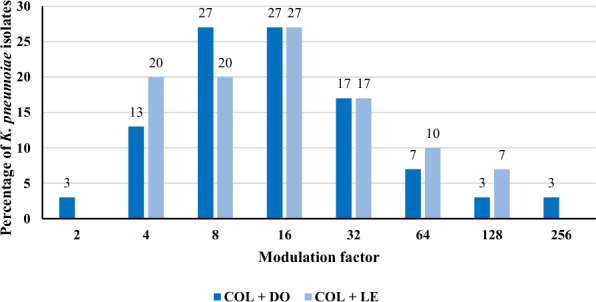
Modulating activity of doxycycline (DO) and levofloxacin (LE), used at subinhibitory concentrations, on the resistance of colistin (COL) among 30 colistin-resistant *K. pneumoniae* isolates

Doxycycline and LE were evaluated for their ability to modify COL resistance across the entire ColRKp collection. Subinhibitory concentrations of DO and LE (0.25 X MIC) were used in this assay. Prior to the assay, the MICs of both antibiotics were determined using the broth microdilution technique against the 30 ColRKp isolates, as presented in the Additional file: Table S6. Both antibiotics demonstrated a 100% resistance-modulating effect when partnered with COL, reducing the MIC of COL by 2 to 256 folds. The highest reductions were achieved when combined with DO and LE, reaching 256 and 128 folds, respectively (Fig. 5 and Additional file: Table S7). Notably, using concentrations equivalent to 0.25 X MIC of DO and LE reversed COL resistance in 80% and 73% of the population, respectively (Additional file: Table S7).

### Survival rates of mice

**Fig. 6:**
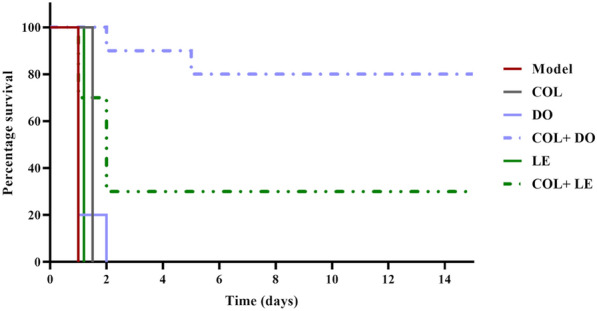
Survival rates of mice in the untreated control (model; 0.9% saline), colistin (COL; 5 mg/kg/day), doxycycline (DO; 50 mg/kg/day), colistin plus doxycycline (COL + DO; 5 mg/kg/day + 50 mg/kg/day), levofloxacin (LE; 150 mg/kg/day), and colistin plus levofloxacin (COL + LE; 5 mg/kg/day + 150 mg/kg/day) groups over a 15-day monitoring period

Mice were i.p. challenged with a 10 X minimal lethal dose of the KP20 isolate. This isolate was chosen for the murine infection model because of its hypermucoid phenotype, pandrug resistance profile, and the highest COL MIC (512 µg/mL) observed among the studied collection. The administration of COL + DO combination therapy significantly enhanced the survival rate of the infected mice compared to the monotherapy or the untreated control groups (Fig. 6; *p*-value < 0.0001). This combination achieved a final survival rate of 80%, demonstrating superior efficacy over COL + LE, which resulted in a 30% survival rate (Fig. 6; *p*-value = 0.0164). Nevertheless, the COL + LE combination showed a significant survival benefit compared with each of the COL, LE, or untreated control groups (*p*-value = 0.0014).

### Organ bioburden

**Fig. 7:**
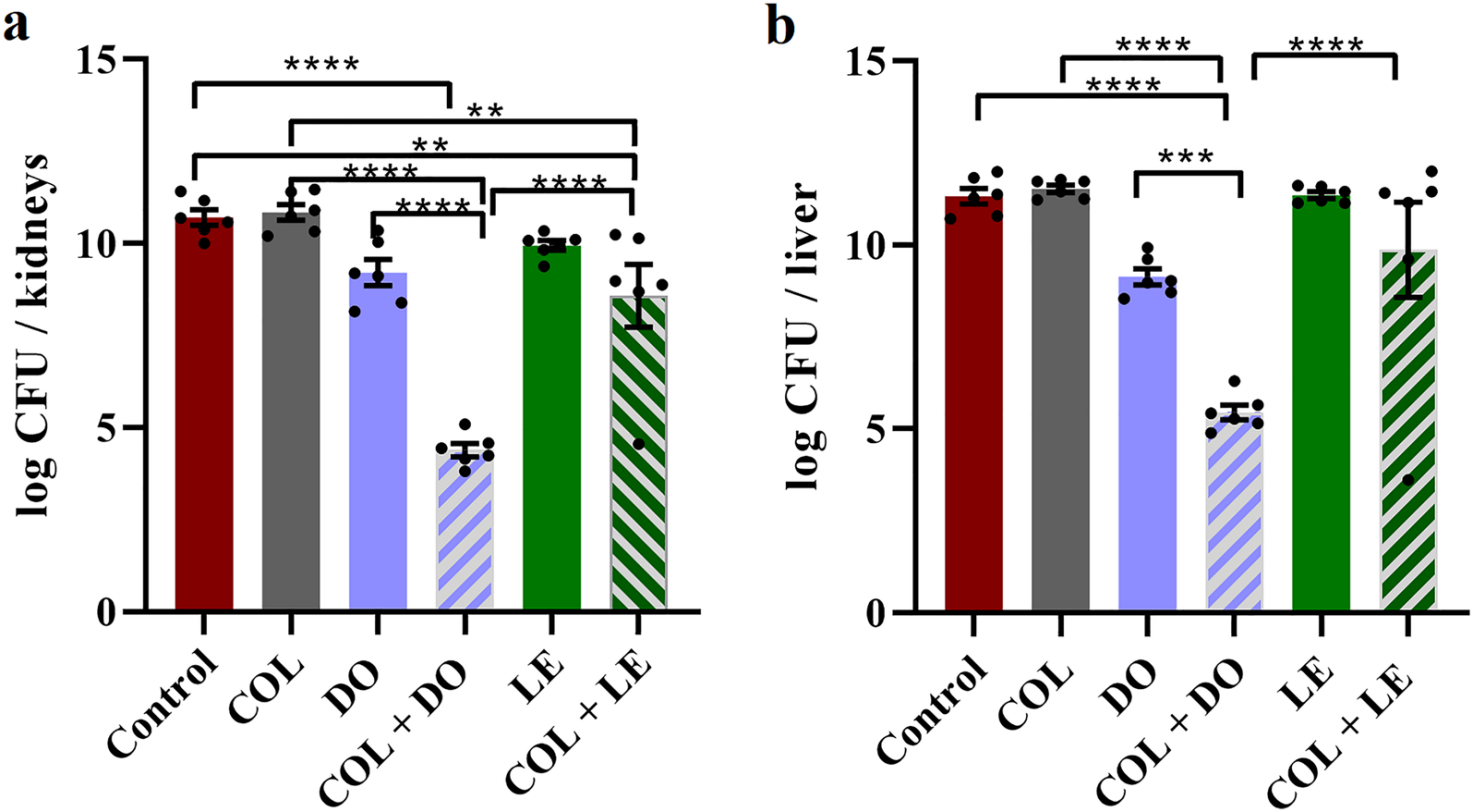
Effects of colistin (COL), doxycycline (DO), levofloxacin (LE), COL + DO, and COL + LE on the bacterial load in the kidneys (**a**) and liver (**b**) of KP20-challenged mice. Error bars depict ± standard error of the mean. Significance levels are denoted as ** for *p*-value ≤ 0.01, *** for *p*-value ≤ 0.001, and **** for *p*-value < 0.0001. One-way analysis of variance (ANOVA) followed by Tukey’s test for post hoc analysis was employed

The mean bacterial count in the kidneys and liver of infected mice treated with COL, DO, and LE did not show significant differences compared to the untreated control group (Fig. 7a and b). However, the bacterial load significantly decreased in the kidneys of mice treated with the COL + DO combination compared to the untreated control (6.32-log reduction), COL-treated (6.45-log reduction), or DO-treated (4.82-log reduction) groups (Fig. 7a; *p*-value < 0.0001). Similarly, the bacterial burden detected in the liver of mice receiving COL + DO was significantly lower by 6.08 logs (*p*-value < 0.0001) and 3.68 logs (*p*-value = 0.007) compared to the COL-treated and DO-treated groups, respectively. On the other hand, the COL + LE combination caused a significant 2.12- and 2.25-log reduction in the bacterial load in the kidneys compared to the untreated control or COL-treated groups, respectively (*p*-value ≤ 0.01), and a non-significant 1.36-log reduction compared to the LE-treated group (*p*-value = 0.19). No significant differences were observed in the bacterial counts in the liver of mice between the COL + LE and the control groups (*p*-value = 0.44), or the COL + LE and the corresponding monotherapy groups. Upon comparing both combinations, COL + DO was superior to COL + LE as it significantly reduced the bioburden by 4.19 and 4.42 logs in the liver and kidneys of infected mice, respectively (*p*-value < 0.0001, Fig. 7a and b).

### Histopathological lung examination

**Fig. 8:**
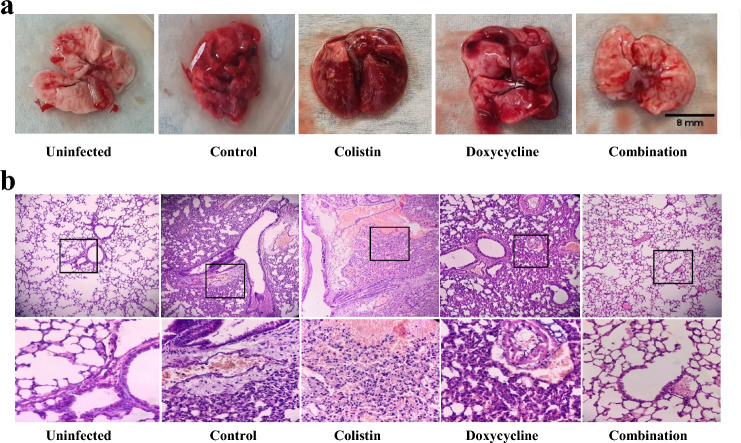
**a** Gross examination of lungs showing a normal appearance with no evidence of inflammatory signs in the uninfected group. In the control, colistin, and doxycycline groups, pulmonary congestion is observed. The colistin-treated group shows a confluence of consolidation areas and a patch formation at the upper left of the lung. The combination group shows a lung appearance closely resembling normal, except for areas of congestion. **b** Histologic findings of the H&E-stained lung sections under a light microscope with the top panels showing X100 magnification followed by an inset at X400 magnification. The uninfected group features normal lung parenchymal tissue, thin-walled alveolar septae, and intact bronchiolar epithelium (X100). Ciliated columnar cells and pneumocytes are lining the alveoli (X400). The control and colistin-treated groups show distorted lung parenchyma, alveolar collapse, blood vessel congestion, and perivascular edema (X100). Destruction of the alveoli replaced by an infiltrate consisting of neutrophils, lymphocytes, and plasma cells is depicted (X400). The doxycycline-treated group displays a relatively preserved architecture of the lung parenchyma, except for the thickened alveolar walls, congested blood vessels, and prominent perivascular edema (X100). The inset reveals inflammatory cellular components, including polymorphs, lymphocytes, histiocytes, and plasma cells (X400). The combination group reveals normal lung parenchymal tissue, except for the mildly congested blood vessels (X100) with an inset showing intact bronchiolar epithelium and thin-walled alveolar septae (X400)

The gross examination of lungs dissected from the COL + DO combination group revealed a close resemblance to the normal lung appearance in the negative control, except for minor areas of congestion, indicating a good response to the dual antimicrobial therapy. In the control group, where mice were challenged with bacteria and received no treatment, the lung featured foci of tan-yellow consolidation surrounded by dark red lung tissue, denoting pulmonary congestion. A similar inflammatory pattern was observed in the COL and DO monotherapy groups (Fig. 8a). H&E-stained lung sections were examined at two magnifications (X100 and X400) under the light microscope (Fig. 8b). Evident blood vessel congestion, prominent perivascular edema, and alveolar collapse were noted in the COL-treated group. In contrast, the architecture of the lung parenchyma was relatively maintained in the DO-treated group, except for the thickened alveolar walls caused by inflammatory components, congested blood vessels, and notable perivascular edema. The bronchiolar epithelium and the alveolar septae in the COL + DO combination group remained intact and resembled normal lung parenchymal tissue. Nonetheless, minor vascular congestion, presumably attributable to a post-inflammatory response, was noted (Fig. 8b).

The degree of inflammation was scored in the lung tissues of the four infected mice groups and recorded as mean values ± standard errors in Table 4. Kruskal–Wallis and Dunn’s multiple comparison tests revealed that the degree of vascular congestion in the COL + DO group was significantly lower than that observed in the control (*p*-value = 0.0009) or the COL-treated (*p*-value = 0.0189) groups. Likewise, the degree of interstitial inflammation was lower in the combination group than that depicted in the control (*p*-value = 0.0352) or the COL-treated (*p*-value = 0.0137) groups. Thus, the dual therapy displayed an apparent decrease in the total inflammatory score compared to the control group (*p*-value < 0.0001).

**Table 4:** Inflammation scoring in lungs of infected mice treated with colistin, doxycycline, or colistin + doxycycline

Scoring of histopathological observation					
	Vascular congestion (0–4)	Alveolar destruction (0–4)	Perivascular edema (0–3)	Interstitial inflammation (0–3)	Total inflammatory score
Control	3 ± 0.37	1.33 ± 0.52	2.67 ± 0.29	1.83 ± 0.29	8.83 ± 0.14
COL^a^	2.5 ± 0.39	1.33 ± 0.52	1.67 ± 0.52	2 ± 0.45	7.5 ± 0.21
DO^b^	1.83 ± 0.53	1.33 ± 0.52	2.83 ± 0.24	1.5 ± 0.55	7.5 ± 0.29
COL + DO	0.67 ± 0.52	1.17 ± 0.53	1.67 ± 0.59	0.83 ± 0.41	4.33 ± 0.61

^a ^*COL* colistin, ^b ^*DO* doxycycline; values are presented as means ± standard error

## Discussion

Infections caused by ColRKp pose significant clinical challenges that can result in prolonged hospitalization, therapeutic impasse, and elevated mortality rates [26]. Understanding the resistance mechanisms and identifying effective treatment strategies are imperative, particularly when these pathogens develop PDR profiles. In the current study, we prospectively collected 30 ColRKp clinical isolates from AMUH, a main referral tertiary hospital in the northern sector of Egypt, and from the laboratories of a private hospital catering to a large proportion of patients through a satellite of eight branches in Alexandria. Both collection sites realistically reflected the situation in a substantial part of the governorate. A systematic review has revealed increasing continental trends of COL resistance among *K. pneumoniae* isolates, with the global incidence rate reaching approximately 12% [16]. Nationally, Zafer et al. reported a frequency of 9.4% for ColRKp in their 2019 study at the National Cancer Institute of Cairo University [50]. In the current study, all ColRKp were recovered from hospitalized patients, with 83.33% of the isolates obtained from the ICUs. The isolates exhibited a high resistance profile, where 17% were PDR, 20% MDR, and 63% displayed an XDR profile. These extremely elevated resistance levels encountered in isolates prevailing in ICUs indicate the intense selective antibiotic pressure exerted in these wards, which serve as hotbeds drawing AMR, surpassing that of other hospital units [51]. Although the collected isolates displayed elevated resistance to almost all tested antibiotics, a susceptibility level of 73% was detected for TGC, designating this glycylcycline antibiotic as a promising alternative for managing ColRKp infections. However, the insufficient absorption of TGC from the gut and its limited urinary excretion represent clinically significant drawbacks [18]. The resistance rates detected in the current study are consistent with those reported in our previous research on ColRKp recovered from patients admitted to ICUs in Alexandria [51]. We believe that the results of both studies could significantly contribute to the development of local antibiograms and provide valuable information that will guide clinicians throughout the hospitals in Alexandria to the most effective empiric antibiotic therapy.

The multidrug resistance detected among the tested ColRKp was associated with a decline in their virulence characteristics, as evidenced by the predominance of non-biofilm formers and non-hypermucoid strains. This may suggest that, while acquiring resistance helps bacterial survival in the presence of antimicrobial pressure, it comes at the cost of bacterial fitness, which tends to reduce the acquisition of multiple virulence traits [52]. Our suggestion coincides well with previous investigations that highlighted the negative correlation existing between antibiotic resistance and biofilm-forming ability [52–54].

The five PDR isolates were chosen for an in-depth genomic analysis, revealing their belonging to three distinct STs: ST14, ST147, and ST383. These STs are recognized as emerging international high-risk clones due to their global distribution across different geographical regions and their association with the PDR phenotype in *K. pneumoniae* reported from various countries: ST14 in Bangladesh [55], Saudi Arabia [56], and the United Arab Emirates [57]; ST147 in Bangladesh [55], the United Arab Emirates [58] and Greece [59]; and ST383 in Qatar [60] and Lebanon [61]. The dissemination of these clones raises concerns as they often carry carbapenemase-encoding genes, such as *bla*_OXA-48_ and *bla*_NDM_ [62–64]. The resistomes of the studied PDR isolates depicted the possession of one or both of these genes, significantly limiting frontline therapy options such as carbapenems or the newly introduced β-lactam–β-lactamase inhibitor combination, CZA, which remains vulnerable against NDM-producing carbapenem-resistant *K. pneumoniae* [65].

Virulome analysis of the PDR isolates revealed the predominance of LPS, *fim*, and *mrk* operons, which are key pathogenicity elements crucial for initiating infection and evading host immune defenses [66]. Additionally, the acquisition of yersiniabactin, aerobactin, or salmochelin siderophore systems [66] further facilitates the establishment of infections by enabling these isolates to thrive in the iron-deficient environment imposed by the host. Notably, *iuc*, known for its pivotal role among the four siderophore systems, was detected in all the PDR isolates [67]. The hypervirulence biomarker, *rmpA*, was present in the hypermucoid strain, KP3, while absent in KP20, a strain expressing hypermucoidy phenotypically. Strains with hypermucoviscous phenotype lacking the *rmpA*/*rmpA2* genes have been previously described, suggesting the involvement of other genetic determinants in expressing this phenotype [68]. Furthermore, the presence of genes conveying resistance to heavy metals in isolates of clinical origin is thought to offer better survival parameters in environments beyond hospital settings [69].

The IncHI1B/IncFIB plasmids carried by the PDR isolates exhibited convergence of AMR, virulence, and heavy metal resistance genes, showcasing their genetic plasticity and promoting the development of bacterial isolates with enhanced infection-producing capabilities [61]. A prior Egyptian study in Alexandria described a hybrid plasmid from a *K. pneumoniae* of the high-risk clone ST383, harboring resistance and virulence genes in a single genetic environment [61]. This plasmid reported in 2022, along with those characterized here, underscores the endemic nature of these vectors, which disseminate pandrug resistance across Egyptian territory [61]. Notably, these megaplasmids were not confined to a specific clone but were rather encountered in isolates from diverse STs (ST14, ST383, and ST147). The abundance of diverse mobile genetic elements in these plasmids likely plays a significant role in their mosaic formation through recurrent genetic transpositions, enhancing their adaptation to bacterial hosts of various clones [70]. Moreover, the resistance loci containing insertion elements can serve as hotspots capturing further resistance genes, thereby creating new resistance pathotypes [71]. If the convergence phenomenon persists, these pathotypes might dominate the currently circulating clones, and since they represent a tremendous treatment challenge, any upcoming onset of hospital infections will be devastating.

While exploring the mechanisms of COL resistance, no plasmid-borne *mcr* genes were detected, suggesting the involvement of chromosomal mutations as key determinants underlying COL resistance in the ColRKp isolates. This finding aligns with previous studies that identified mutations in chromosomal loci as the primary drivers of COL resistance [50, 51, 72]. Among these, alterations in *mgrB* were the most frequently encountered in the WGS-characterized PDR isolates. MgrB, a negative feedback regulator of the PhoP/PhoQ two-component system, inhibits the activation of phosphorylated PhoP [72]. This inhibition reduces the PhoPQ signal transduction to *arnBCADTEF* and *pmrCAB* operons, thereby decreasing the production of L-Ara4N and pEtN [72]. Consequently, genetic changes in *mgrB* lead to upregulating PhoPQ and PmrAB two-component systems linked through PmrD. This upregulation promotes the synthesis of LPS-modifying molecules and confers resistance to COL [73]. A chromosomal mutation of G109A within *mgrB* was detected in an ST383 isolate, resulting in a deleterious amino acid substitution of G37S in MgrB. This mutation was identical to one observed in our previous study, belonging to a ColRKp isolate of the same ST, K-, and O-types [51]. This suggests clonal expansion and cross-transmission of ColRKp isolates within different Alexandrian hospitals. The non-sense mutation detected at C88T in *mgrB*, known to generate a truncated non-functional MgrB, has been previously reported in Italy [74], Iran [75], India [76], Norway [77], Brazil [78], China [79], France, and Colombia [80]. Expression of a dysfunctional MgrB is expected in the KP11 isolate, with its *mgrB* split via insertional inactivation by IS*Kpn14* at a nucleotide position 35 [81]. Previous complementation assays using wild-type *mgrB* successfully restored COL susceptibility in isolates bearing mutated *mgrB* at C88T and G109A or disrupted *mgrB* by insertional inactivation [79, 80, 82], providing strong evidence that these mutational events are directly related to COL resistance. Additionally, we identified a deleterious substitution within PhoQ, E397G, known to affect PhoQ phosphorylation due to its proximity to the ATP-binding region, a position of paramount importance [83]. Besides the previously reported alterations in *mgrB* and PhoQ, our study detected an unprecedented deleterious mutation, A200Y, in CrrB*.* The two-component regulatory system, CrrAB, stands for “colistin resistance regulation”, and is composed of *crrB*, expressing a histidine kinase, and its adjacent response regulator, *crrA* [84]. This newly identified amino acid substitution lies at a critical region, within the putative histidine kinase domain of CrrB, suggesting a potential impact on its function and a postulated mechanism conferring resistance to COL [85]. Moreover, mutations in *crrB* were speculated to trigger the expression of CrrC, a connector protein, resulting in the upregulation of *pmrHFIJKLM* operon and *pmrC*, and a decreased susceptibility to COL [85]. Notably, CrrAB was entirely missing in ST14 and ST383 isolates, although its absence is not documented to be linked with COL resistance [86], as its presence varies among different *K. pneumoniae* strains [84]. Additional alterations observed in PmrC, ArnB, and ArnT reflected the varied mutation patterns adopted by the tested ColRKp isolates and corroborated several previous reports [86, 87]. Nonetheless, the impact of these mutations on COL resistance remains unclear and merits further inquiry and validation.

The treatment of infections caused by ColRKp imposes a major challenge to clinicians, as most available antibiotics become ineffective when used as monotherapy against these life-threatening pathogens. Combination antimicrobial therapy thus becomes a reliable therapeutic strategy to mitigate the tolling crisis of pandrug resistance [26]. We evaluated the COL-containing combinations using the checkerboard experiment against the five PDR isolates. Seven antibiotics were selected to represent different classes, mechanisms of action and based on previously reported synergy with COL [18, 26]. We discovered that COL functioned synergistically with IPM and MRP against 40% and 60% of tested isolates, respectively; with TIG, CIP, and SXT against 80%; and with DO and LE against 100% of the isolates. Notably, the COL + DO and COL + LE combinations reversed COL resistance, achieving susceptibility levels of 2 µg/mL, categorizing both combinations as promising. Furthermore, when combined with COL, the MICs of DO dropped below its breakpoint in 80% of the isolates, exhibiting what is known as “clinically relevant synergy”. Clinically relevant synergy, a concept that surfaced nowadays in the literature, describes combinations where the concentrations of both antibiotics fall within the susceptible or intermediate range at ΣFICI ≤ 0.50 [22]. Inspired by these results, we sought to provide evidence that the reversal of COL resistance by DO or LE was not merely a sporadic strain-dependent incidence but could be observed across the entire ColRKp collection. At their subinhibitory concentrations, DO and LE acted as COL-resistance-modifying agents, reversing COL resistance in 80% and 73% of the population, respectively. Previous researchers [18, 88, 89] examining the synergistic efficacy of COL + DO or COL + LE combinations in Gram-negative bacteria explained that COL tends to increase the entry-to-exit ratio of antibiotics with which it acts in synergy by exerting a subinhibitory permeabilizing impact on the bacterial outer membrane. By compromising the membrane integrity, COL ensures accelerated intracellular accumulation of the second antibiotic, reaching sufficient levels to initiate its mechanism of action through the inhibition of protein, in the case of DO, or DNA synthesis in the case of LE [18, 88, 89].

The efficacy of the promising combinations was further assessed in vivo using a murine infection model. The COL + DO combination demonstrated superiority over COL + LE, rescuing 80% of the infected animals compared to 30% after a 15-day monitoring period. In addition, the survival rates observed in COL + DO significantly surpassed those obtained for the untreated or monotherapy groups. In correlation with survival rate results, the COL + DO combination showed an excellent effect while enumerating the bacterial load in the liver and kidneys of infected mice, decreasing the bioburden by 4.19 and 4.42 logs, respectively, relative to the COL + LE combination, and the differences were significant at *p*-value < 0.0001. Moreover, the potential efficacy of COL + DO combination therapy against ColRKp is substantiated by the promising histopathological results detected in this study. The severely inflamed lungs showing congestion in infected mice displayed a close-to-normal lung appearance upon treatment with the COL + DO combination. The combination decreased lung inflammation and preserved a nearly intact bronchial epithelium and unimpaired alveolar space. We believe this is the first study to assess the in vivo efficacy of the COL + DO combination against ColRKp using a murine model. The most relevant study we could reclaim reported the efficiency of polymyxin B + DO combination against *Pseudomonas aeruginosa* lung infections [89].

We acknowledge the limitations of this study. We examined only a small number of isolates obtained from a single governorate. Despite providing in vitro data from the checkerboard array and resistance modulation assay, the study did not track the time-kill kinetics. Notwithstanding these limitations, the study yielded an important key strength; the in vitro resistance modulation assay investigated both COL + DO and COL + LE combinations on 30 ColRKp isolated from clinical settings. Moreover, the in vivo murine bacteremia model revealed the predictive clinical value for both regimens. Therefore, our study could be considered a foundational step toward initiating more extensive national and international surveillance research.

## Conclusions

In conclusion, we have characterized PDR ColRKp isolates, providing genomic insights into their clones, resistomes, and virulomes. The hybrid IncHI1B/IncFIB plasmids carried by these isolates, converging resistance and virulence, threaten the spread of superbugs that can cause serious infections unresponsive to broad-spectrum antibiotics. In addition, we have detailed various genetic mechanisms conferring resistance to COL. Nonetheless, our study offers a solution to reverse this COL resistance and reestablish the role of COL as a last-resort antibiotic. The combination of COL with DO represents the first demonstration of a combination that effectively reaches pertinent clinical levels of COL susceptibility in vitro, while in vivo increases mice survival rates, decreases organ bioburden, and preserves lung histology. Future research is warranted to assess the efficacy of the COL + DO combination against a larger number of clinical isolates to provide a holistic picture of its potential benefits for ColRKp infections. Clinical validation and further exploration of pharmacokinetic properties are fundamental before implementing these regimens into clinical practice.

## Supplementary Information

Additional file 1.

## Data Availability

The WGS dataset of the present study was submitted to NCBI under BioProject number PRJNA1063361 with sample accession numbers (SAMN39335492 - SAMN39335496) which correspond to KP3, KP8, KP11, KP20, and KP22 isolates. https://www.ncbi.nlm.nih.gov/bioproject/?term=PRJNA1063361, Plasmids designated pEGY_KP3, pEGY_KP8, pEGY_KP11, pEGY_KP20, and pEGY_KP22 can be found at NCBI database with GenBank accessions CP154635 to CP154639, respectively. https://www.ncbi.nlm.nih.gov/search/all/?term=CP154635, https://www.ncbi.nlm.nih.gov/search/all/?term=CP154636, https://www.ncbi.nlm.nih.gov/search/all/?term=CP154637, https://www.ncbi.nlm.nih.gov/search/all/?term=CP154638, https://www.ncbi.nlm.nih.gov/search/all/?term=CP154639.

## Abbreviations

ColRKp: Colistin-resistant *K. pneumoniae*
*mcr*: Mobile colistin resistance gene
LPS: Lipopolysaccharide
CLSI: Clinical Laboratory Standards Institute
MLST: Multilocus sequencing typing
AMR: Antimicrobial resistance
mini-BAL: Mini-bronchoalveolar lavage
ST: Sequence type
MDR: Multidrug-resistant
XDR: Extensively drug-resistant
PDR: Pandrug-resistant
WT: Wild-type
ESBL: Extended-spectrum β-lactamases
