# Supplementary material for: Efficacy of colistin-based combinations against pandrug-resistant whole-genome-sequenced Klebsiella pneumoniae isolated from hospitalized patients in Egypt: an in vitro/vivo comparative study
Source: Gut Pathog. 2024 Dec 3;16:73. doi: 10.1186/s13099-024-00667-z (PMC11616336; doi:10.1186/s13099-024-00667-z)
Supplement: Supplementary file 1 — Additional file 1. [file 13099_2024_667_MOESM1_ESM.pdf]

Supplementary Information

Table S1 The sequence of primer pairs used to detect mobile colistin resistance genes (*mcr*), their expected product sizes, and PCR annealing conditions.

Target gene	Nucleotide sequence (5'⟶3')	Annealing temperature (˚C)	Amplicon size (bp)	Reference
mcr-1	F:5'-CGGTCAGTCCGTTTGTTC-3'<br>R:5'-CTTGGTCGGTCTGTAGGG-3'	51.5	309	[1]
mcr-3	F:5'-TTGGCACTGTATTTTGCATTT-3'<br>R:5'-TTAACGAAATTGGCTGGAACA-3'	51.5	542	[2]
mcr-7	F:5'-TTATCAACCGCTGGGGACTG-3'<br>R:5'-GCTTGATCTCGATGTTGGGCAC-3'	58	377	[3]
mcr-8	F:5'-AACCGCCAGAGCACAGAATT-3'<br>R:5'-TTCCCCCAGCGATTCTCCAT-3'	56.1	667	[4]

Table S2 Demographic data and clinical origin of the collected colistin-resistant *Klebsiella pneumoniae* isolates.

Isolate code	Source[a]	Date of collection	Hospital unit[b]	Clinical origin[c]	Gender	Age[d]
KP1	Private hospital labs	01/2021	ICU	Mini-BAL	Male	83 Y
KP2	Private hospital labs	01/2021	Ward	Sputum	Female	13 Y
KP3	Private hospital labs	01/2021	ICU	Wound	Male	58 Y
KP4	Private hospital labs	01/2021	ICU	Blood	Male	18 Y
KP5	AMUH	01/2021	ICU	Urine	Male	75 Y
KP6	Private hospital labs	02/2021	ICU	Urine	Male	45 Y
KP7	Private hospital labs	02/2021	Ward	Urine	Male	46 Y
KP8	AMUH	02/2021	ICU	Mini-BAL	Female	65 Y
KP9	Private hospital labs	02/2021	Ward	Blood	Male	54 Y
KP10	AMUH	02/2021	Ward	-	-	-
KP11	Private hospital labs	02/2021	ICU	Blood	Male	23 Y
KP12	Private hospital labs	02/2021	ICU	Mini-BAL	Female	62 Y
KP13	Private hospital labs	03/2021	ICU	Blood	Female	67 Y
KP14	Private hospital labs	04/2021	ICU	Blood	Female	-
KP15	Private hospital labs	05/2021	ICU	Mini-BAL	Male	55 Y
KP16	AMUH	05/2021	Ward	Urine	Male	67 Y
KP17	AMUH	05/2021	ICU	Sputum	Male	60 Y
KP18	AMUH	05/2021	ICU	Pus	Male	71 Y
KP19	Private hospital labs	05/2021	ICU	Wound	Male	35 Y
KP20	Private hospital labs	05/2021	ICU	Urine	Male	80 Y
KP21	Private hospital labs	05/2021	ICU	CVC	Female	-
KP22	Private hospital labs	05/2021	ICU	Wound	Female	8 Y
KP23	Private hospital labs	06/2021	NICU	Blood	-	3 D
KP24	Private hospital labs	06/2021	ICU	MiniBAL	Male	63 Y
KP25	Private hospital labs	06/2021	ICU	MiniBAL	Male	92 Y
KP26	Private hospital labs	06/2021	ICU	Blood	Male	-
KP27	Private hospital labs	06/2021	NICU	Blood	-	16 D
KP28	Private hospital labs	07/2021	NICU	Blood	-	15 D
KP29	Private hospital labs	07/2021	NICU	Blood	-	20 D
KP30	Private hospital labs	07/2021	NICU	Blood	-	7 D

[a]AMUH: Alexandria Main University Hospital; [b]ICU: intensive care unit; NICU: neonatal intensive care unit; [c]BAL: bronchoalveolar lavage; CVC: central venous catheter; [d]Y: years; D: days; -: unknown

Table S3 Minimum inhibitory concentration (MIC) of colistin, resistance profile, treatment options, and resistance status of the tested *K. pneumoniae* isolates.

Isolate code	Colistin MIC (µg/mL)	Resistance Profile[a]	Treatment options	Resistance status[b]
KP1	16	AMC, P/T, CAZ, CZA, CTR, CPM, AT, IPM, MRP, ETP, AK, **DO**, **TGC**, CIP, LE	GEN, AZ, and SXT	XDR
KP2	256	AMC, P/T, CAZ, CZA, CTR, CPM, AT, IPM, MRP, ETP, GEN, AK, AZ, **DO**, TGC, CIP, LE	SXT	XDR
KP3	16	AMC, P/T, CAZ, CZA, CTR, CPM, AT, IPM, MRP, ETP, GEN, AK, AZ, DO, TGC, CIP, LE, SXT	-	PDR
KP4	64	AMC, P/T, CAZ, CZA, CTR, CPM, AT, IPM, MRP, ETP, GEN, AK, AZ, CIP, LE, SXT	DO and TGC	XDR
KP5	32	AMC, P/T, CAZ, CZA, CTR, CPM, AT, IPM, MRP, ETP, GEN, AK, AZ, **DO**, CIP, LE, SXT	TGC	XDR
KP6	32	AMC, P/T, CAZ, CZA, CTR, CPM, AT, IPM, MRP, ETP, GEN, AK, AZ, DO, CIP, LE, SXT	TGC	XDR
KP7	32	AMC, P/T, CAZ, CZA, CTR, CPM, AT, IPM, MRP, ETP, GEN, AK, AZ, **DO**, CIP, LE	TGC and SXT	XDR
KP8	8	AMC, P/T, CAZ, CZA, CTR, CPM, AT, IPM, MRP, ETP, GEN, AK, AZ, DO, TGC, CIP, LE, SXT	-	PDR
KP9	64	AMC, P/T, CAZ, CTR, CPM, AT, IPM, MRP, ETP, GEN, AK, AZ, DO, CIP, LE, SXT	CZA and TGC	XDR
KP10	16	AMC, P/T, CAZ, CTR, CPM, AT, MRP, **ETP**, GEN, CIP, LE, SXT	CZA, IPM, AK, AZ, DO, and TGC	MDR
KP11	128	AMC, P/T, CAZ, CZA, CTR, CPM, AT, IPM, MRP, ETP, GEN, AK, AZ, DO, TGC, CIP, LE, SXT	-	PDR
KP12	32	AMC, P/T, CAZ, CZA, CTR, CPM, IPM, MRP, ETP, GEN, AK, AZ, DO, TGC, CIP, LE, SXT	AT	XDR
KP13	32	AMC, P/T, CAZ, CZA, CTR, CPM, AT, IPM, MRP, ETP, GEN, AK, AZ, DO, CIP, LE, SXT	TGC	XDR
KP14	64	AMC, P/T, CAZ, CTR, CPM, AT, IPM, MRP, ETP, GEN, AK, AZ, DO, CIP, LE, SXT	CZA and TGC	XDR
KP15	8	AMC, P/T, CAZ, CZA, CTR, CPM, AT, IPM, MRP, ETP, GEN, AK, AZ, DO, CIP, LE, SXT	TGC	XDR
KP16	32	AMC, P/T, CAZ, CZA, CTR, CPM, AT, IPM, MRP, ETP, GEN, AK, AZ, DO, CIP, LE, SXT	TGC	XDR

Table S3 continued.

Isolates	Colistin MIC (µg/ml)	Resistance Profile[a]	Treatment options	Resistance status[b]
KP17	64	AMC, P/T, CAZ, CZA, CTR, CPM, AT, IPM, MRP, ETP, GEN, AK, AZ, CIP, LE, SXT	DO and TGC	XDR
KP18	16	AMC, P/T, CAZ, CPM, IPM, MRP, ETP, GEN, AK, AZ, DO, CIP, LE	CZA, CTR, AT, TGC, and SXT	MDR
KP19	64	AMC, P/T, CAZ, CTR, CPM, IPM, MRP, ETP, GEN, AK, AZ, DO, CIP, LE, SXT	CZA, AT, and TGC	XDR
KP20	512	AMC, P/T, CAZ, CZA, CTR, CPM, AT, IPM, MRP, ETP, GEN, AK, AZ, DO, TGC, CIP, LE, SXT	-	PDR
KP21	32	AMC, P/T, CAZ, CTR, CPM, AT, IPM, MRP, ETP, GEN, AK, AZ, **DO**, CIP, LE, SXT	CZA and TGC	XDR
KP22	16	AMC, P/T, CAZ, CZA, CTR, CPM, AT, IPM, MRP, ETP, GEN, AK, AZ, **DO**, **TGC**, CIP, LE, SXT	-	PDR
KP23	16	AMC, P/T, CAZ, CZA, CTR, CPM, AT, IPM, MRP, ETP, **GEN**, CIP, LE, SXT	AK, AZ, DO, and TGC	MDR
KP24	16	AMC, P/T, CAZ, CZA, CTR, CPM, AT, IPM, MRP, ETP, GEN, AK, AZ, DO, CIP, LE, SXT	TGC	XDR
KP25	32	AMC, P/T, CAZ, CZA, CTR, CPM, AT, IPM, MRP, ETP, GEN, AK, AZ, DO, CIP, LE, SXT	TGC	XDR
KP26	8	AMC, P/T, CAZ, CZA, CTR, CPM, AT, IPM, MRP, ETP, GEN, AK, AZ, DO, CIP, LE, SXT	TGC	XDR
KP27	4	AMC, P/T, CAZ, CZA, CTR, CPM, IPM, MRP, ETP, GEN, AK, AZ, CIP, LE, SXT	AT, DO, and TGC	MDR
KP28	16	AMC, P/T, CAZ, CZA, CTR, CPM, AT, IPM, MRP, ETP, GEN, AK, AZ, CIP, LE, SXT	DO and TGC	XDR
KP29	64	AMC, P/T, CAZ, CZA, CTR, CPM, IPM, MRP, ETP, AZ, CIP, LE, SXT	AT, GEN, AK, DO, and TGC	MDR
KP30	32	AMC, P/T, CAZ, CZA, CTR, CPM, AT, IPM, MRP, ETP, **GEN**, CIP, LE, SXT	AK, AZ, DO, and TGC	MDR

[a]AMC amoxicillin/clavulanate; P/T piperacillin/tazobactam; CZA ceftazidime/avibactam; CAZ ceftazidime; CTR ceftriaxone; CPM cefepime; AT aztreonam; IPM imipenem; MRP meropenem; ETP ertapenem; GEN gentamicin; AK amikacin; AZ azithromycin; DO doxycycline; TGC tigecycline; CIP ciprofloxacin; LE levofloxacin; SXT sulfamethoxazole/trimethoprim; [b]MDR multidrug-resistant; XDR extensively drug-resistant; PDR pandrug-resistant. The bold format indicates the intermediate susceptibility of the isolate to the tested antibiotic.

Table S4 Assembly statistics of the whole-genome-sequenced pandrug-resistant *K. pneumoniae* isolates.

Isolate code	Total length	Total number of contigs	Largest contig	GC (%)	N50 [a] (bp)	N75 [b] (bp)	L50[c]	L75[d]
KP3	5,787,376	223	392184	56.59	120283	64664	16	31
KP8	5,857,559	292	310142	56.59	94027	39110	22	44
KP11	5,911,136	452	230119	56.74	76606	32344	24	51
KP20	6,031,286	255	289667	56.53	151136	57582	15	32
KP22	6,041,885	326	296421	56.57	106549	46226	18	37

[a] N50: Length of the contig at the midpoint of contigs arranged from largest to smallest to covering at least 50% of the total base content of the assembly; [b] N75: Length at which 75% of the assembly length is contained in the contigs of that size or larger; [c] L50: Minimal number of contigs that contains half the total base content of the assembly; [d] L75: Minimal number of contigs that covers at least 75% of the total assembly length.

Table S5 Chromosomal point mutations conferring resistance to cephalosporins, carbapenems, and fluoroquinolones in the whole-genome-sequenced pandrug-resistant *K. pneumoniae* isolates.

Isolate code	Point mutations conferring resistance to:		
	**Cephalosporins**	**Carbapenems**	**Fluoroquinolones**
KP3	*ompK36* (N49S, L59V, T184P)	*ompK37* (I70M, I128M)	*acrR* (P161R, G164A, F172S, R173G, L195V, F197I, K201M), *gyrA* (S83F, D87N), and *parC* (S80I)
KP8	*ompK36* (N49S, L59V, T184P)	*ompK37* (I70M, I128M)	*acrR* (P161R, G164A, F172S, R173G, L195V, F197I, K201M), *gyrA* (S83F, D87N), and *parC* (S80I)
KP11	*ompK36* (N49S, L59V, L191S, F207W, D224E, L228V, E232R, T254S)	*ompK36* (A217S, N218H) and *ompK37* (I70M, I128M)	*acrR* (P161R, G164A, F172S, R173G, L195V, F197I, K201M)
KP20	*ompK36* (N49S, L59V, G189T, F198Y, F207Y, T222L, D223G, E232R, N304E)	*ompK36* (A217S) and *ompK37* (I70M, I128M)	*acrR* (P161R, G164A, F172S, R173G, L195V, F197I, K201M), *gyrA* (S83I), and *parC* (S80I)
KP22	*ompK36* (N49S, L59V, G189T, F198Y, F207Y, T222L, D223G, E232R, N304E)	*ompK36* (A217S) and *ompK37* (I70M, I128M)	*acrR* (P161R, G164A, F172S, R173G, L195V, F197I, K201M), *gyrA* (S83I), and *parC* (S80I)

Table S6 Minimum inhibitory concentration values for doxycycline and levofloxacin against 30 *K. pneumoniae* isolates.

Isolate code	Doxycycline MIC (µg/mL)	Levofloxacin MIC (µg/mL)	Isolate code	Doxycycline MIC (µg/mL)	Levofloxacin MIC (µg/mL)
KP1	8	64	KP16	64	32
KP2	8	128	KP17	4	64
KP3	32	64	KP18	16	64
KP4	4	32	KP19	16	64
KP5	8	128	KP20	32	128
KP6	16	128	KP21	8	128
KP7	8	128	KP22	8	128
KP8	32	128	KP23	4	8
KP9	16	128	KP24	32	128
KP10	4	32	KP25	32	128
KP11	128	8	KP26	128	32
KP12	16	128	KP27	4	256
KP13	64	64	KP28	4	256
KP14	16	32	KP29	4	2
KP15	32	128	KP30	2	4

Table S7 Modulation of colistin resistance by subinhibitory concentrations of doxycycline and levofloxacin in 30 colistin-resistant *K. pneumoniae* isolates.

Isolate code	MIC (µg/mL)		Modulation Factor
KP1	COL[a] alone	16	-
	COL at ¼ MIC DO[b]	2	8
	COL at ¼ MIC LE[c]	4	4
KP2	COL alone	256	-
	COL at ¼ MIC DO	2	128
	COL at ¼ MIC LE	2	128
KP3	COL alone	16	-
	COL at ¼ MIC DO	2	8
	COL at ¼ MIC LE	1	16
KP4	COL alone	64	-
	COL at ¼ MIC DO	2	32
	COL at ¼ MIC LE	4	16
KP5	COL alone	32	-
	COL at ¼ MIC DO	8	4
	COL at ¼ MIC LE	2	16
KP6	COL alone	32	-
	COL at ¼ MIC DO	2	16
	COL at ¼ MIC LE	2	16
KP7	COL alone	32	-
	COL at ¼ MIC DO	2	16
	COL at ¼ MIC LE	2	16
KP8	COL alone	8	-
	COL at ¼ MIC DO	2	4
	COL at ¼ MIC LE	2	4
KP9	COL alone	64	-
	COL at ¼ MIC DO	4	16
	COL at ¼ MIC LE	1	64
KP10	COL alone	16	-
	COL at ¼ MIC DO	1	16
	COL at ¼ MIC LE	4	4
KP11	COL alone	128	-
	COL at ¼ MIC DO	2	64
	COL at ¼ MIC LE	2	64
KP12	COL alone	32	-
	COL at ¼ MIC DO	4	8
	COL at ¼ MIC LE	1	32
KP13	COL alone	32	-
	COL at ¼ MIC DO	4	8
	COL at ¼ MIC LE	2	16

Table S7 continued.

Isolate code	MIC (µg/mL)		Modulation Factor
KP14	COL alone	64	-
	COL at ¼ MIC DO	1	64
	COL at ¼ MIC LE	2	32
KP15	COL alone	8	-
	COL at ¼ MIC DO	1	8
	COL at ¼ MIC LE	1	8
KP16	COL alone	32	-
	COL at ¼ MIC DO	8	4
	COL at ¼ MIC LE	4	8
KP17	COL alone	64	-
	COL at ¼ MIC DO	2	32
	COL at ¼ MIC LE	4	16
KP18	COL alone	16	-
	COL at ¼ MIC DO	1	16
	COL at ¼ MIC LE	2	8
KP19	COL alone	64	-
	COL at ¼ MIC DO	8	8
	COL at ¼ MIC LE	2	32
KP20	COL alone	512	-
	COL at ¼ MIC DO	2	256
	COL at ¼ MIC LE	4	128
KP21	COL alone	32	-
	COL at ¼ MIC DO	1	32
	COL at ¼ MIC LE	1	32
KP22	COL alone	16	-
	COL at ¼ MIC DO	2	8
	COL at ¼ MIC LE	2	8
KP23	COL alone	16	-
	COL at ¼ MIC DO	1	16
	COL at ¼ MIC LE	4	4
KP24	COL alone	16	-
	COL at ¼ MIC DO	1	16
	COL at ¼ MIC LE	2	8
KP25	COL alone	32	-
	COL at ¼ MIC DO	1	32
	COL at ¼ MIC LE	0.5	64
KP26	COL alone	8	-
	COL at ¼ MIC DO	2	4
	COL at ¼ MIC LE	2	4

Table S7 continued.

Isolate code	MIC (μg/mL)		Modulation Factor
KP27	COL alone	4	-
	COL at ¼ MIC DO	2	2
	COL at ¼ MIC LE	1	4
KP28	COL alone	16	-
	COL at ¼ MIC DO	2	8
	COL at ¼ MIC LE	1	16
KP29	COL alone	64	-
	COL at ¼ MIC DO	2	32
	COL at ¼ MIC LE	8	8
KP30	COL alone	32	-
	COL at ¼ MIC DO	2	16
	COL at ¼ MIC LE	2	16

[a] COL: colistin; [b] DO: doxycycline; [c] LE: levofloxacin

References

1. Liu YY, Wang Y, Walsh TR, Yi LX, Zhang R, Spencer J, et al. Emergence of plasmid-mediated colistin resistance mechanism MCR-1 in animals and human beings in China: a microbiological and molecular biological study. Lancet Infect Dis. 2016;16(2):161-8.
2. Yin W, Li H, Shen Y, Liu Z, Wang S, Shen Z, et al. Novel plasmid-mediated colistin resistance gene *mcr-3* in *Escherichia coli*. mBio. 2017;8(3).
3. Azam M, Gaind R, Yadav G, Sharma A, Upmanyu K, Jain M, et al. Colistin resistance among multiple sequence types of *Klebsiella pneumoniae* is associated with diverse resistance mechanisms: a report from India. Front Microbiol. 2021;12:609840.
4. Salloum T, Panossian B, Bitar I, Hrabak J, Araj GF, Tokajian S. First report of plasmid-mediated colistin resistance *mcr-8.1* gene from a clinical *Klebsiella pneumoniae* isolate from Lebanon. Antimicrob Resist Infect Control. 2020;9(1):94.
